# Prevalence and nutritional quality of free food and beverage acquisitions at school and work by SNAP status

**DOI:** 10.1371/journal.pone.0257879

**Published:** 2021-10-13

**Authors:** Aviva A. Musicus, Anne N. Thorndike, Jason P. Block, Eric B. Rimm, Sara N. Bleich

**Affiliations:** 1 Department of Social and Behavioral Sciences, Harvard T.H. Chan School of Public Health, Boston, Massachusetts, United States of America; 2 Department of Medicine, Massachusetts General Hospital, Boston, Massachusetts, United States of America; 3 Harvard Medical School, Boston, Massachusetts, United States of America; 4 Department of Population Medicine, Harvard Medical School, Boston, Massachusetts, United States of America; 5 Department of Nutrition, Harvard T.H. Chan School of Public Health, Boston, Massachusetts, United States of America; 6 Department of Health Policy and Management, Harvard T.H. Chan School of Public Health, Boston, Massachusetts, United States of America; Pennington Biomedical Research Center, UNITED STATES

## Abstract

**Background:**

The dual burden of poor diet quality and food insecurity makes free food—food acquired at no cost—a very important part of the nutrition safety net for low-income families. The goal of this study was to determine the national prevalence and nutritional quality of free food acquired separately in two settings: 1) by children at school; and 2) by employees at work; both stratified by participation in the Supplemental Nutrition Assistance Program (SNAP).

**Methods:**

Using National Household Food Acquisition and Purchase Survey data (2012; n = 4,826 U.S. households containing 5,382 employed adults and 3,338 school-aged children), we used survey-weighted proportions to describe free food acquisition and linear regression to compare the 2010 Healthy Eating Index (HEI-2010) for free/non-free food acquisition events (i.e., meals) by SNAP status. Analyses were conducted in 2019–2020.

**Results:**

SNAP households had more free acquisition events (29.6%) compared to non-SNAP households (<185% federal poverty level (FPL) = 22.3%; ≥185%FPL = 21.0%, p’s<0.001). For SNAP-participant children, free acquisition events at school had a higher mean HEI-2010 compared to non-free acquisition events at school (50.3 vs. 43.8, p = 0.033) and free acquisition events by SNAP-non-participant children ≥185%FPL at school (50.3 vs. 38.0, p = 0.001). Free and non-free acquisition events at work had relatively low HEI-2010s, with no differences by SNAP status.

**Conclusions:**

Over one fifth of all food acquisition events were free, but free food acquisitions at school and work were relatively unhealthy. For children participating in SNAP, free food acquired at school had higher nutritional quality. Improving the dietary quality of free foods could improve the health of families, especially those participating in SNAP.

## Introduction

The average American diet consistently falls short of national dietary guidelines, which contributes to negative health outcomes such as cardiovascular disease and type 2 diabetes [1, 2]. Low-income Americans are even less likely to meet national guidelines [2] and experience higher rates of diet-related chronic diseases [3]. Food insecurity, defined as a lack of consistent access to enough food for an active, healthy life, is also associated with negative health outcomes [4] and is more than three times higher among Americans living in poverty compared to the general population (35.3% vs 11.1%) [5]. This dual burden of poor diet quality and food insecurity makes free food—food and beverages acquired at no cost to the consumer, with costs borne by some other entity (e.g., friends, workplaces, government)—a very important part of the nutrition safety net for low-income families.

A recent report by the United States Department of Agriculture (USDA) found that one fifth of all food and beverages in the American diet are acquired at no cost, with higher rates of free food acquisition by participants in the Supplemental Nutrition Assistance Program (SNAP) [6]. SNAP is the largest program in the federal nutrition safety net, helping 38 million Americans, nearly half of whom are children, afford food each month [7]. SNAP participants receive monthly funds to pay for groceries; thus, foods purchased with SNAP benefits were not considered “free” in the USDA report. Instead, much of the free food the report identified was acquired at school or at work. Half of all food and beverage acquisitions at schools were free, largely due to the National School Lunch Program (NSLP), the second largest federal nutrition assistance program, which provides free and reduced-price lunches to approximately 30.4 million children daily [8]. About half of NSLP participants also live in households that receive SNAP [9, 10]. Children in SNAP households are automatically enrolled to receive free school meals, while all children in the nation’s highest-poverty school districts receive free school meals regardless of their household income through the Community Eligibility Provision [9]. Meals provided through the NSLP are required to adhere to strict nutrition standards based on the *Dietary Guidelines for Americans* (DGA) [11]. In contrast, while over 70 percent of workplace food acquisitions were free from sources such as catered lunch meetings [6], most workplaces do not have standardized nutrition requirements, and there are generally few nutritional standards governing food offered in institutional settings [11, 12].

Free food in institutional settings is of particular interest from a public health perspective, as institutional-level policies could improve the nutritional quality of free foods provided. Cost is a major barrier to consumption of healthier foods [13], especially among low-income households, so the provision of free food offers a unique opportunity to modify food preferences by allowing people to try new foods without any monetary risk; this may be an especially effective tactic to encourage children to learn to enjoy more nutritious foods, as studies have shown that children need repeated exposure to unfamiliar foods before accepting them [13]. Healthier free food in institutions could also promote healthy eating as normative behavior [14].

Despite the available evidence that free food makes up a considerable portion of total food acquired by U.S. households, it is unknown whether acquisition and nutritional quality of free food vs. non-free food is similar across the population or if it varies by participation in SNAP. To answer this important question, we focused on two institutional settings in which a large proportion of free food is acquired—schools and workplaces. Specifically, this study examined the national prevalence and nutritional quality of free food acquired separately: 1) by children at school; and 2) by employees at work; both stratified by household SNAP participation.

## Methods

### Study sample

Data were obtained from the National Household Food Acquisition and Purchase Survey (FoodAPS, 2012), a nationally representative survey of 4,826 U.S. households representing 14,317 individuals, released in 2015 by the USDA as a restricted-use data set [15]. FoodAPS collected data on all food acquisitions and purchases (including food at home and away from home) by all members of sampled households over one week, with oversampling of lower-income and SNAP households. The primary household meal planner provided demographic and diet- and health-related information about the household and its members through two in-person interviews and three brief telephone interviews. Each household member ≥11 years old was asked to track all food acquisition events (i.e., meals) by using food diaries, scanning barcodes, and saving receipts from stores and restaurants. Adults used their own food diaries to record foods acquired by children under age 11 in their household [15]. FoodAPS respondents recorded information about each acquisition event, including the cost, timing of the meal (breakfast, lunch, dinner, snack, multiple meals), all food items acquired (e.g., sandwich, milk, apple), and location. These acquisition events included both “food at home”, food and drinks that were brought into the home and used to prepare meals for consumption at home or elsewhere (e.g., bread and meat purchased at a grocery store later used to make a sandwich to bring to work), and “food away from home”, food and drinks acquired and consumed away from home (e.g., a sandwich purchased at a work cafeteria). Multi-part incentives were offered to encourage participation by all household members. Analysis for this study occurred in 2019–2020.

### Measures

The study’s primary outcomes were the prevalence and nutritional quality of free and non-free food and beverage acquisition events separately at school and work, stratified by SNAP status. The term “free food” is used to indicate an acquisition event in which all items (foods and/or beverages) were acquired for free. Examples of free food in the FoodAPS dataset include groceries from food pantries, a restaurant meal paid for by a household member’s friend, or a meal cooked and served at a friend’s house. An example of non-free food in the FoodAPS dataset would be a meal cooked and served in the household, as the food would have been acquired at the supermarket by a household member and that member would have designated which household members shared that food. Examples of free food acquired by a school-aged child at school include a NSLP school lunch if the child qualified for free school lunch (e.g., slice of cheese pizza, side salad, canned peaches, apple juice, and ice cream), or a cupcake acquired in class during a birthday celebration. Examples of non-free food acquired by a school-aged child at school include a hamburger and French fries purchased from the cafeteria by a child that does not qualify for free school lunch, or a bag of pretzels purchased from a vending machine. Free food acquired by employees at work could include coffee and a doughnut or a catered lunch (e.g., sandwich, bag of chips, cookie). Non-free food at work includes food and beverages purchased at a work cafeteria or vending machine. FoodAPS solely contains acquisition data, so consumption details are unknown.

Nutritional quality was measured using the 2010 Healthy Eating Index (HEI-2010), which measures alignment with the 2010 DGA using a density approach (e.g., food groups and nutrients per 1000 calories and the fatty acid ratio), and has been widely used to assess and compare diet quality [2, 16, 17]. The total score is comprised of 12 components and has a maximum score of 100. The HEI increases with consumption of 9 dietary components encouraged in the 2010 DGA (e.g., whole grains) and decreases with consumption of 3 dietary components recommended in moderation (e.g., refined grains) [16]. The 12 individual component scores were examined as secondary outcomes. HEI-2010 total and component scores were calculated based on previously described methods using the composition of acquired foods in the FoodAPS database, which include descriptions and sizes from the Food and Nutrient Database for Dietary Studies [18]. Other secondary outcomes included the 10 most commonly acquired foods and beverages (category 2 level of USDA’s “What We Eat in America” categories [19], e.g., sandwiches) for free at school and at work. The 10 most commonly acquired free school foods were additionally examined, stratified by whether students received free school lunch.

School outcomes were evaluated among school-aged individuals 5–18-years-old, and work outcomes were evaluated among any household member that reported working at a job or business (all ≥16 years old). Individuals were divided across three categories according to SNAP status, based on FoodAPS designations: 1) SNAP participants, 2) lower-income SNAP-eligible non-participants (household incomes <185% FPL, “SNAP eligible non-participants”), and 3) higher-income participants that were SNAP ineligible (household incomes ≥185% FPL, “SNAP ineligible”). These household income cutoffs are consistent with prior USDA [2, 6] and peer-reviewed FoodAPS analyses [20]. Household income was self-reported, and SNAP participation status in the prior 30 days was determined by both survey responses and matches to state administrative records.

### Statistical analysis

Analyses were conducted using Stata 15.1 software. Differences in characteristics of school-aged and employed individuals and their households by SNAP/income groups were tested using survey-weighted linear and logistic regression for continuous and categorical variables respectively. Survey-weighted means and proportions were used to describe the percentage of total free food and beverage acquisition events during the sampled week by SNAP status among three population groups: all households, school-aged individuals, and employed individuals. Percentages of free food acquisition events for children at school and for employees at work were also examined. Survey-weighted linear and logistic regression was used to respectively compare the means and proportions of free acquisition events by SNAP status within each population group. Survey-weighted means were used to examine HEI-2010 total and component scores for free and non-free food and beverage acquisition events for children at school and for employees at work by SNAP status. Scores were compared using survey-weighted linear regression with an interaction term for whether food was acquired for free and SNAP status, and adjusted for individuals’ age, sex, race (white, black, other), Hispanic ethnicity, household number of children 5–18 years old, household WIC status, and household food insecurity (food secure or insecure [low or very low food security as measured by the USDA’s Adult Food Security Scale]). Analyses of employed individuals at work additionally controlled for education (≤high school, some college, or ≥college) and marital status (married or unmarried).

We conducted two sensitivity analyses examining primary outcomes using modified income cutoffs for household SNAP status (non-participants ≤130% FPL and >130% FPL—households must generally be at or below this threshold to qualify for SNAP [7]) and restricting the school HEI analyses to children that received free school lunch.

## Results

### Free food acquisition at school

Demographic characteristics of school-aged individuals and their households are shown by SNAP status in Table 1. Compared to SNAP ineligible individuals, school-aged SNAP participants were significantly younger, and a higher proportion were Hispanic and Black. Their households were significantly larger and more food insecure. Compared to their SNAP non-participant counterparts, a significantly higher proportion of school-aged SNAP participants received free school lunch through the NSLP.

**Table 1:** Characteristics of school-aged (5–18 years old) individuals and their households by SNAP/income groups.

**School-aged individual characteristics**	**SNAP participants** (n = 1508)	**non-SNAP *<*185% FPL** (n = 788)	**non-SNAP ≥185% FPL** (n = 1042)
Mean Age (SD)	10.9 (6.4)	11.7 (5.3)^a^	11.4 (3.1)^a^
Male (%)	51.7	47.8	49.2
Hispanic (%)	33.8	32.4	15.2^b^
Race (%)			
White	52.8	65.4^a^	76.0^b^
Black	30.5	17.5^a^	10.9^b^
Other	16.7	17.1	13.1
School in session during study period (%)	58.4	52.9	58.7
School Level (%)			
Kindergarten	7.5	4.7^a^	3.6^a^
Elementary/Primary	36.3	25.5^a^	28.9^a^
Middle School/Junior High	16.0	17.7	16.3
High School	16.2	25.6^a^	22.9^a^
Other	1.1	3.1	1.2
Cost of school lunch (%)			
Free	93.2	59.4^b^	17.1^b^
Reduced Price	3.4	23.2^b^	12.3^a^
Full Price	3.4	17.4^b^	70.6^b^
**Characteristics of households with school-aged individuals**	**SNAP HH** (n = 783)	**non-SNAP <185% FPL** (n = 418)	**non-SNAP** **≥185% FPL** (n = 624)
Mean number of food acquisition events per week (SD)	16.3 (16.5)	16.7 (14.8)	18.1 (8.5)^a^
Mean number of household members (SD)	4.4 (2.9)	4.4 (2.6)	4.0 (1.0)^b^
Mean number of household children (<19 years old) (SD)	2.4 (2.2)	2.3 (1.8)	1.9 (0.8)^b^
Mean number of school-aged household children (5–18 years old) (SD)	1.9 (1.9)	1.9 (1.5)	1.7 (0.8)^a^
Anyone in household receiving WIC (%)	55.1	43.3	13.1^b^
Food insecure (low or very low, 30-day, adult) (%)	42.5	40.8	7.0^b^

School-aged individuals are individuals aged 5–18 years old. Food insecurity was measured with USDA’s 10-question 30-day Adult Food Security Scale (e.g., “In last 30 days, worried food would run out before we got more money”, “Couldn’t afford to eat balanced meals in last 30 days”). “Often” or “Sometimes” = 1; “Never” = 0. Raw score 3–5 = Low food security; 6–10 = very low food security. “Other School Level” includes “other school” and “home-schooled”. Total school level does not add up to 100 because other children were classified as “on vacation”, “not old enough”, etc.

^a^ significantly different from SNAP, p<0.05 (survey-weighted linear/logistic regression for continuous/categorical variables).

^b^ significantly different from SNAP, p<0.001 (survey-weighted linear/logistic regression for continuous/categorical variables).

**Fig 1:**
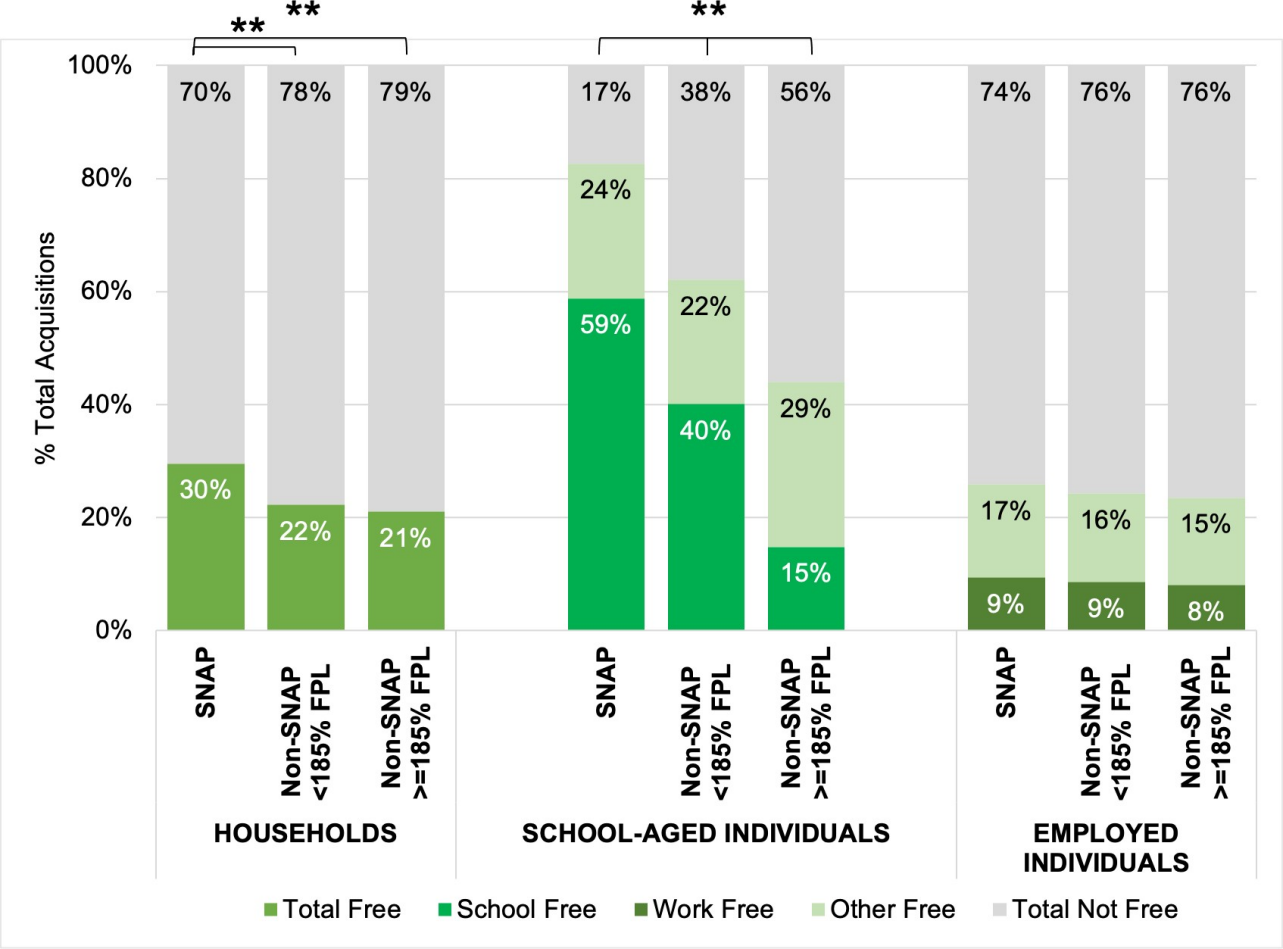
Proportions of free and non-free acquisition events across SNAP status by population group. Survey-weighted proportions compared with survey-weighted logistic regression within population groups. ** % free significantly different between SNAP/income status groups, p<0.001.

Fig 1 shows proportions of free and non-free food and beverage acquisition events by SNAP status within each population group. Overall, a significantly higher percentage of SNAP households’ acquisition events were free (29.6%) compared to SNAP non-participant households (eligible: 22.3%, p<0.001) and ineligible households (21.0%, p<0.001). For SNAP-participant children, 82.6% of acquisition events were free, and more than half of those free events were at school (58.8% of all acquisition events). This was a significantly higher proportion of free food acquisition events overall compared to SNAP-non-participant children (p<0.001), for whom free acquisition events made up 62.1% (SNAP eligible) and 44.0% (SNAP ineligible) of all food acquisition events. SNAP-non-participant children also acquired much of their free food while at school (eligible: 40.1% acquisition events; ineligible: 14.8%).

**Fig 2:**
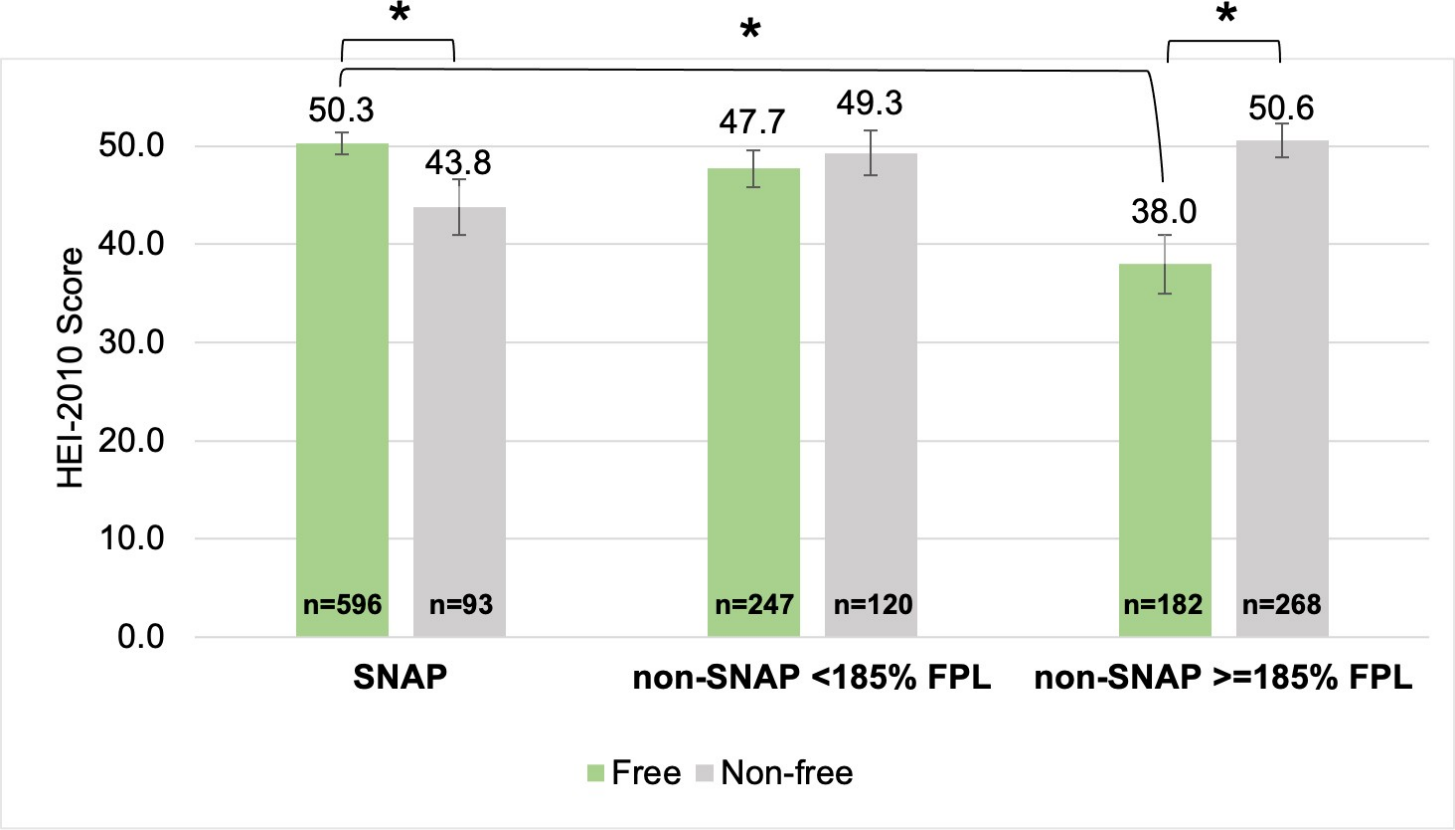
Mean HEI-2010 scores of free vs. non-free food acquisition events by children at school, by SNAP status. Survey-weighted linear regression, adjusted for individuals’ age, sex, race, Hispanic ethnicity, household number of children 5–18, household food insecurity, and household WIC status. Error bars represent SEs. *Sig. dif. p<0.05.

Fig 2 shows adjusted mean HEI-2010 scores of free and non-free food acquisition events by children at school. SNAP-participant children’s free food acquisition events at school had a higher HEI-2010 compared to their non-free food acquisition events at school (50.3 vs. 43.8, p = 0.033) and compared to SNAP-ineligible children’s free food acquisition events at school (50.3 vs. 38.0, p = 0.001). SNAP-ineligible children’s free food acquisition events at school had a lower HEI-2010 compared to their non-free food acquisition events at school (38.0 vs. 50.6, p = 0.004). When analyses were restricted to free acquisition events among children who received free school lunch, there were no significant differences in HEI-2010 (range: 47.7–51.4) (S1 Fig).

The HEI component score analysis of free food acquisition events at school (S1 Table) showed that SNAP-participant children had significantly higher component scores for total vegetables, greens and beans, total fruit, whole fruit, total protein foods, seafood and plant protein, and empty calories, and a significantly lower component score for sodium compared to SNAP-ineligible children. There were no significant differences between the two SNAP status categories for whole grains, dairy, fatty acids ratio, or refined grains. S2 and S3 Tables show the most common foods and beverages children acquired for free at school; across all children, these included fruit, plain and flavored milk, 100% juice, sandwiches, vegetables, white potatoes, and pizza. Sweet bakery products and savory snacks made up a larger proportion of free acquisitions for children that did not receive free school lunch compared to those that did.

### Free food acquisition at work

Demographic characteristics of employed individuals and their households are shown by SNAP status in Table 2. Compared to SNAP ineligible individuals, employed SNAP participants were significantly younger, and a higher proportion were Hispanic and Black. Their households were significantly larger and more food insecure. Compared to their SNAP non-participant counterparts, a significantly lower proportion of employed SNAP participants were married and had attended college.

**Table 2:** Characteristics of employed individuals (≥16 years old) and their households by SNAP/income groups.

**Employed individuals’ characteristics**	**SNAP participants** (n = 1347)	**non-SNAP <185% FPL** (n = 1131)	**non-SNAP ≥185% FPL** (n = 2904)
Mean Age (SD)	37.1 (19.8)	39.9 (17.7)^a^	43.0 (10.2)^b^
Male (%)	47.1	51.6	52.9^a^
Hispanic (%)	32.4	30.2	11.6^b^
Race (%)			
White	56.3	67.3^b^	78.9^b^
Black	25.5	14.6^b^	9.0^b^
Other	18.2	18.1	12.2^a^
Education (%)			
High school or less	63.0	51.8^b^	27.9^b^
Some college	27.4	36.2^a^	31.3
College+	8.6	11.9	40.6^b^
Married (%)	29.2	44.8^b^	56.8^b^
**Characteristics of households with employed individuals**	**SNAP HH** (n = 935)	**non-SNAP <185% FPL** (n = 750)	**non-SNAP** **≥185% FPL** (n = 1680)
Mean number of food acquisition events per week (SD)	14.2 (14.3)	12.3 (11.9)^a^	13.4 (6.6)
Mean number of household members (SD)	3.8 (2.9)	3.0 (2.6)^b^	2.6 (1.1)^b^
Mean number of household children (<19 years old) (SD)	1.6 (2.4)	1.1 (1.9)^b^	0.7 (0.8)^b^
Mean number of school-aged household children (5–18 years old) (SD)	1.0 (2.0)	0.8 (1.6)^a^	0.5 (0.7)^b^
Anyone in household receiving WIC (%)	60.6	40.9^a^	13.5^b^
Food insecure (low or very low, 30-day, adult) (%)	39.3	31.4^a^	7.5^b^

Employed individuals are any individuals who reported working at a job or business. Food insecurity was measured with USDA’s 10-question 30-day Adult Food Security Scale (e.g., “In last 30 days, worried food would run out before we got more money”, “Couldn’t afford to eat balanced meals in last 30 days”). “Often” or “Sometimes” = 1; “Never” = 0. Raw score 3–5 = Low food security; 6–10 = very low food security.

^a^ significantly different from SNAP, p<0.05 (survey-weighted linear/logistic regression for continuous/categorical variables).

^b^ significantly different from SNAP, p<0.001 (survey-weighted linear/logistic regression for continuous/categorical variables).

**Fig 3:**
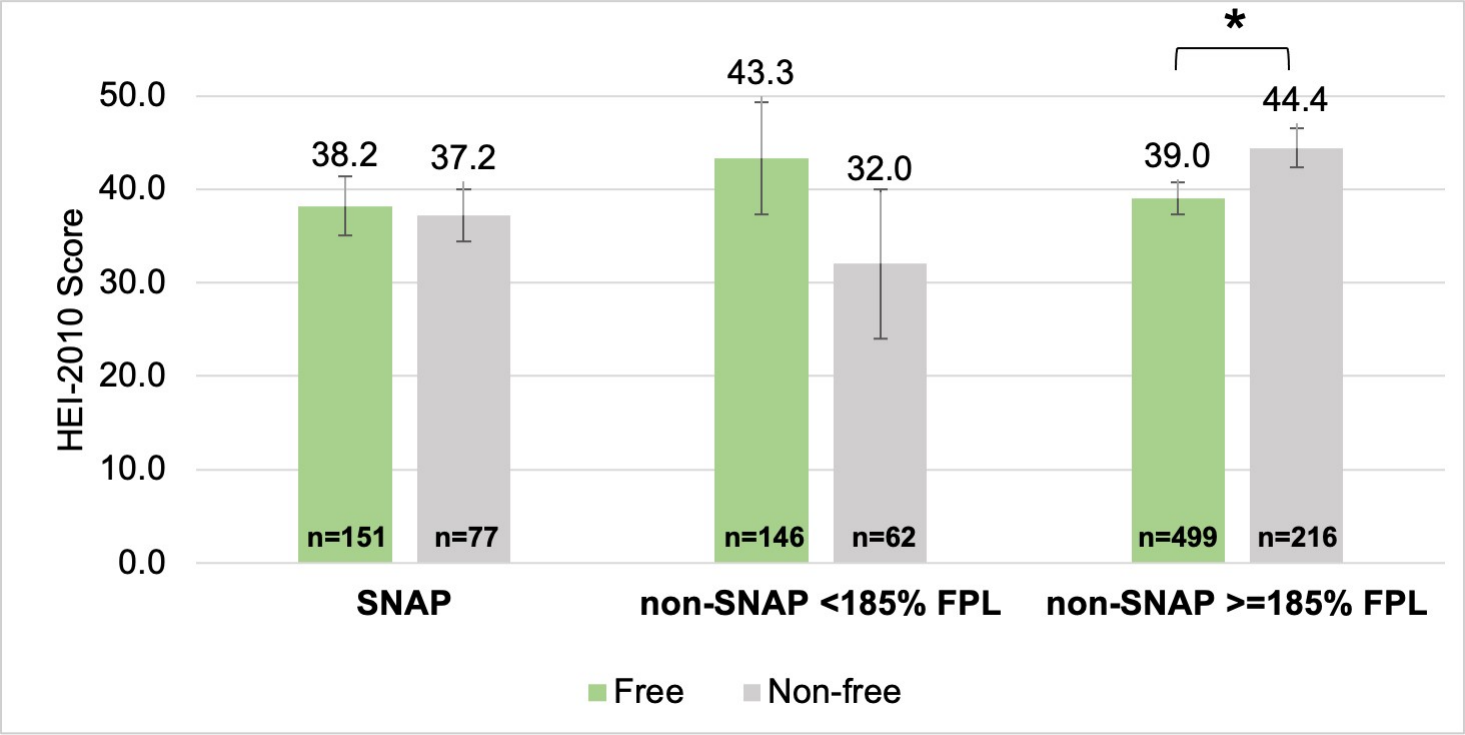
Mean HEI-2010 scores of free vs. non-free food acquisition events by employees at work, by SNAP status. Survey-weighted linear regression, adjusted for individuals’ age, sex, race, Hispanic ethnicity, education, marriage status, household number of children 5–18, household food insecurity, and household WIC status. Error bars represent SEs. *Sig. dif. p<0.05.

Only 24–26% of employed individuals’ total food acquisition events were free, and there were no significant differences by SNAP status (Fig 1). Of all their acquisition events, 8–9% were free and at work. SNAP-ineligible employees’ free food acquisition events at work had a significantly lower HEI-2010 compared to their non-free food acquisition events at work (39.0 vs. 44.4, p = 0.025) (Fig 3). There were no other significant differences within or between SNAP status, but HEI-2010 scores were consistently low for free and non-free work acquisition events (32.0–44.4) compared to the maximum score possible, 100.

There were no significant differences in HEI component scores of SNAP-participant employees’ free food acquisition events at work compared to those of SNAP-ineligible employees at work (S4 Table). The most common free foods and beverages employees acquired at work included coffee and tea, sandwiches, sweetened beverages, vegetables, and water (S5 Table). Sweet bakery products made up a larger proportion of free food for SNAP non-participants compared to SNAP participants.

Primary outcomes analyzed with a modified income cutoff for household SNAP status (non-participants ≤130% FPL and >130% FPL) did not differ significantly from main analyses (S2–S4 Figs).

## Discussion

Although much research has been devoted to overall diet quality of lower-income populations, this is the first study to evaluate the nutritional quality of free and non-free food in a large nationally representative sample of adults and children and to examine differences by SNAP participation. We found that 30% of all SNAP households’ food acquisition events were free, which was significantly higher than the proportion of SNAP-ineligible households’ food acquisition events that were free (21%). Our results indicate that SNAP participants relied on free food more than higher-income non-participants. One explanation for this is that current SNAP benefit levels are too low for many households, and others face limited SNAP eligibility and barriers to participation [21]. Addressing these issues could diminish the overall need for free food among SNAP participants. At the same time, increasing the nutritional quality of free food in schools and workplaces represents an important opportunity to improve dietary intake and health, particularly for low-income families participating in food assistance programs.

### Free food acquisition at school

The majority (83%) of total acquisition events for children in SNAP households were free, and more than half of their total food was acquired for free at school. Although the overall dietary quality of children’s school food acquisition events was low compared to the maximum possible HEI score, SNAP-participant children’s free school food acquisition events were of higher nutritional quality compared to their non-free food acquisition events at school and compared to SNAP-ineligible children’s free food acquisition events at school. Our finding that children who received free school lunch had higher HEI-2010 scores at school regardless of SNAP status suggests the NSLP may improve diet quality, especially among SNAP participants, as they are categorically eligible for the program. Other studies have found that the NSLP has led to better diet quality for low-income children compared to higher-income children [22–26].

NSLP nutrition standards were updated in 2012 for the first time in more than 15 years with the passage of the Healthy Hunger-Free Kids Act (HHFKA) [27]. By July 1, 2012, schools were required to offer meals with more fruit, vegetables, and whole grains, while reducing the amount of calories, sodium, saturated fat, and added sugars [27]. Furthermore, the 2014 implementation of the Smart Snacks in School regulation required higher nutrition standards for all foods sold during the school day (e.g., vending machines) [28]. Several attempts have been made to weaken these requirements. Although a federal court struck down a 2018 rule that weakened nutrition standards for sodium and whole grains [29], the USDA re-proposed the rule allowing "flexibilities for milk, whole grains, and sodium requirements" in November 2020 [30].

FoodAPS acquisition data were collected while USDA was phasing in the updated 2012 HHFKA school meal standards. FoodAPS nutrient composition data for school foods, however, were collected from 2009–2010. Therefore, our findings reflect the nutrition content of school foods before the new standards were implemented. It is thus likely that if acquisition and nutrient composition data were collected today—or even if FoodAPS nutrient composition data were contemporaneous with its acquisition data—the free food that SNAP participants acquired at school could have an even higher HEI-2010 score due to product reformulation (e.g., lower-sodium bread), and due to full implementation of school meal standards across the U.S. Indeed, studies using more recent data have documented an increase in diet quality due to the updated nutrition standards [31, 32]. For example, researchers found that HHFKA standards substantially increased the effects of the NSLP on the diet quality of higher-income children, from the NSLP having no effect on their diet quality pre-HHFKA to the NSLP increasing their diet quality by 4.4% post-HHFKA [32]. They also found that the NSLP improved diet quality for low-income students by 6.8% before the HHFKA and by 10.6% after the HHFKA [32]; although this difference was not significantly different, it does suggest that these standards moderately improved low-income children’s diets. While this study relied on 24-hour recalls, other researchers have employed more direct consumption measurements via plate waste to document the effects of the HHFKA’s implementation, and found significant increases in diet quality for low-income children [31]. Furthermore, another study found that the HHFKA’s implementation was associated with a significant decline in obesity for children in poverty, suggesting that the new standards have had benefits for low-income children beyond improved diet quality [33]. Given that the majority of U.S. public school students now qualify for free and reduced-price school meals [34], and that low-income students are at the highest risk for obesity [35], our findings combined with those of more recent studies suggest that the NSLP’s 2012 nutritional standards should be maintained, and not weakened.

Aside from school meals, our analyses also focused on the nutritional quality of free foods acquired throughout the school day. Policies that govern nutrition standards for these foods could be leveraged to increase the nutritional quality of free food at school for everyone. School wellness policies were first federally mandated in 2004 for schools participating in federal school meal programs and were updated by the HHFKA [36]. We found that much of the free school foods acquired outside of the school lunch program included sweet bakery products (e.g., cookies, brownies) and savory snacks. Although these items made up a smaller proportion of SNAP participants’ overall free school food, they were prevalent across all groups. As these foods are often served during parties, distributing non-food alternatives or limiting which foods can be distributed for celebrations and as classroom rewards could improve the nutritional quality of free food at school for all children [37]; preliminary evidence suggests that such wellness policies can have beneficial outcomes for obesity prevention [38].

### Free food acquisition at work

This study also highlighted the lower nutritional quality of foods provided for free at workplaces across income levels. Some of the most commonly acquired free foods and beverages included sandwiches, sweetened beverages, and sweet bakery products, which often have high levels of added sugar and sodium. Workplace wellness policies with strong nutrition standards could improve the nutritional quality of free foods offered at work. More than half of all U.S. states have laws related to workplace wellness programs, although few directly address diet at work [39]. Procurement policies can also be implemented to improve diet quality at work, as has been done in numerous worksites across the country at the national, state, and local level [12, 40].

### Limitations

This study had several limitations. Our data solely contain acquisition, not consumption, information. However, acquisitions still provide insight into what institutions are providing and individuals are likely to be eating. The FoodAPS dataset has some general limitations that have been discussed elsewhere [41], such as underreporting of acquisitions over the course of the week due to response fatigue, and the potential for parents to underreport acquisitions made by their under-11 children. Because FoodAPS allowed respondents to enter in food from multiple meals without identifying which food corresponded to which meal, we were unable to examine whether free foods like “sweet bakery products” were acquired during lunch or as part of a celebratory snack. While this study only examined schools and workplaces, the nutritional quality of free food provided at other sites, such as food pantries, is also of interest. In a recent FoodAPS analysis, researchers found that free food from food pantries was of higher nutrient quality than non-free food among households receiving charitable food assistance [42]. Future research should gather new data on free and non-free food acquisition and consumption at school, work, and other institutions like food pantries to understand how the nutritional quality of free offerings may have changed since FoodAPS data was collected.

## Conclusions

Free food makes up a large portion of food acquired by U.S. households, and represents the majority of food acquired by children living in families receiving SNAP benefits. For these children, free food acquisition events at school had higher nutritional quality, but the overall nutritional quality of free food acquisition events at school and work was relatively low. Policy efforts by governments (e.g., nutrition standards for school meals) and the private sector (e.g., institutional wellness policies) can help to both improve the health profile of free food and reduce access to less healthy foods, which can in turn improve overall dietary quality for U.S. families.

## Supporting information

S1 Fig Sensitivity analysis: Mean HEI-2010 scores of free food acquisitions by children at school by SNAP status, restricted to children who reported receiving free school lunch. Survey-weighted, adjusted for individuals’ age, sex, race, Hispanic ethnicity, household number of children 5–18, household food insecurity, and household WIC status. No significant differences.
(TIF)

S2 Fig Sensitivity analysis: Free and non-free acquisitions by location across SNAP status (cutoff 130% FPL), survey-weighted proportions. * % free significantly different between groups, p<0.05; ** p≤0.001.
(TIF)

S3 Fig Sensitivity analysis: Mean HEI-2010 scores of free vs. non-free food acquisitions by children at school, by SNAP status (cutoff 130% FPL). Survey-weighted, adjusted for individuals’ age, sex, race, Hispanic ethnicity, household number of children 5–18, household food insecurity, and household WIC status. *Sig. dif. p<0.05.
(TIF)

S4 Fig Sensitivity analysis: Mean HEI-2010 scores of free vs. non-free food acquisitions by employees at work, by SNAP status (cutoff 130% FPL). Survey-weighted, adjusted for individuals’ age, sex, race, Hispanic ethnicity, education, marriage status, household number of children 5–18, household food insecurity, and household WIC status. No significant differences.
(TIF)

S1 Table HEI-2010 component density scores of free food acquisitions by children at school. Survey-weighted, adjusted for individuals’ age, sex, race, Hispanic ethnicity, household number of children 5–18, household food insecurity, and household WIC status. ^a^ Significantly different from SNAP individuals, p<0.05. ^b^ Calories from solid fats, alcohol, and added sugars; threshold for counting alcohol is >13 grams/1000 kcal.
(DOCX)

S2 Table Most commonly acquired foods and beverages for free by children at school. Survey-weighted, % out of total foods and beverages acquired for free at school by school-aged individuals.
(DOCX)

S3 Table Most commonly acquired foods and beverages for free by children at school, stratified by whether child receives free school lunch. Survey-weighted, % out of total foods and beverages acquired for free at school by school-aged individuals who do/don’t receive free school lunch.
(DOCX)

S4 Table HEI-2010 component densities of free food acquisitions by employees at work. Survey-weighted, adjusted for individuals’ age, sex, race, Hispanic ethnicity, education, marriage status, household number of children 5–18, household food insecurity, and household WIC status. ^a^ Significantly different from SNAP individuals, p<0.05. ^b^ Calories from solid fats, alcohol, and added sugars; threshold for counting alcohol is >13 grams/1000 kcal.
(DOCX)

S5 Table Most commonly acquired foods and beverages for free by employees at work. Survey-weighted, % out of total foods and beverages acquired for free at work by employed individuals.
(DOCX)
