# Supplementary figures and images for: Prevalence and nutritional quality of free food and beverage acquisitions at school and work by SNAP status
Source: PLoS One. 2021 Oct 13;16(10):e0257879. doi: 10.1371/journal.pone.0257879 (PMC8514130; doi:10.1371/journal.pone.0257879)

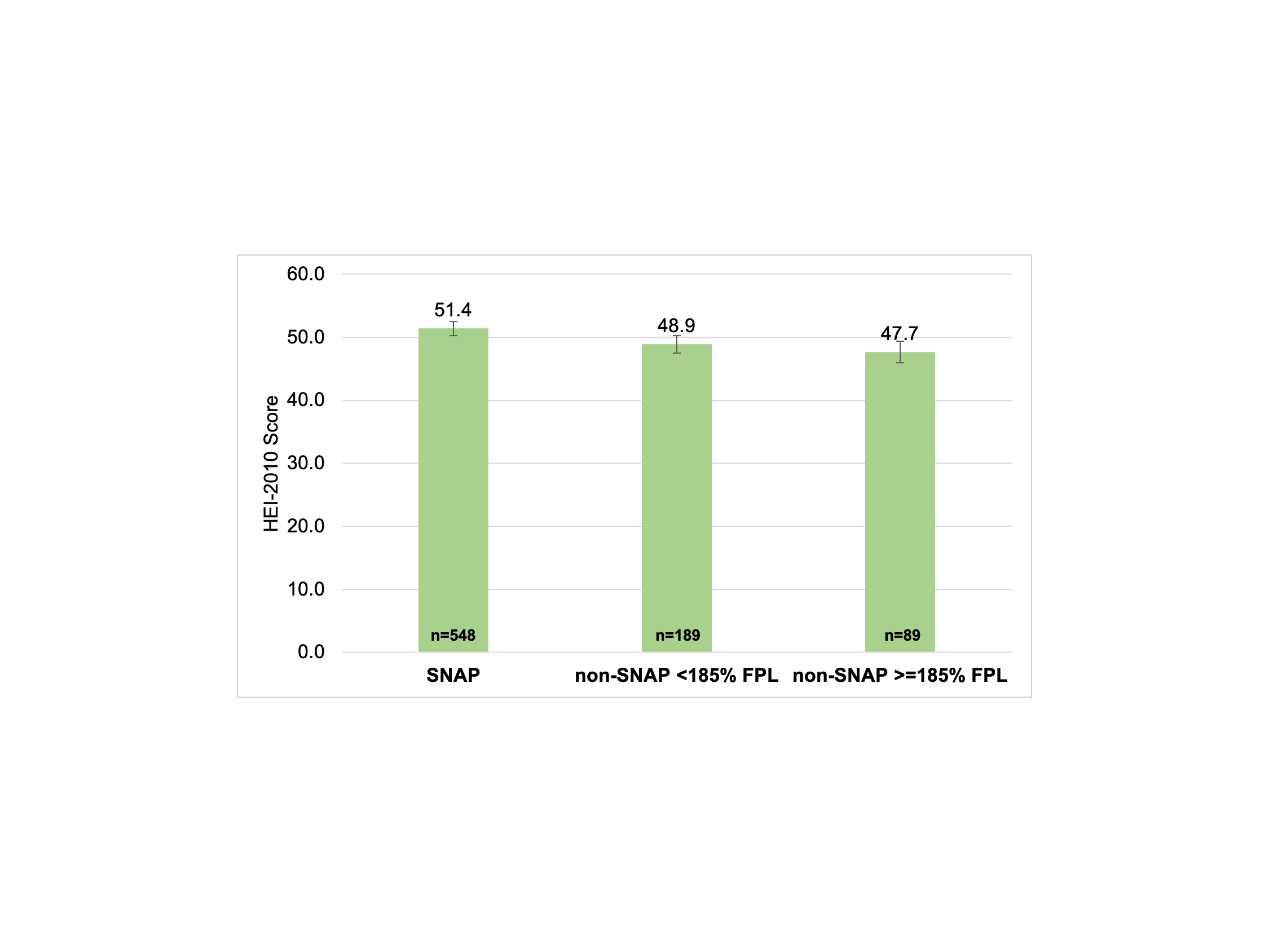

Supplement: S1 Fig — Survey-weighted, adjusted for individuals’ age, sex, race, Hispanic ethnicity, household number of children 5–18, household food insecurity, and household WIC status. No significant differences. (TIF) [file pone.0257879.s001.tif]

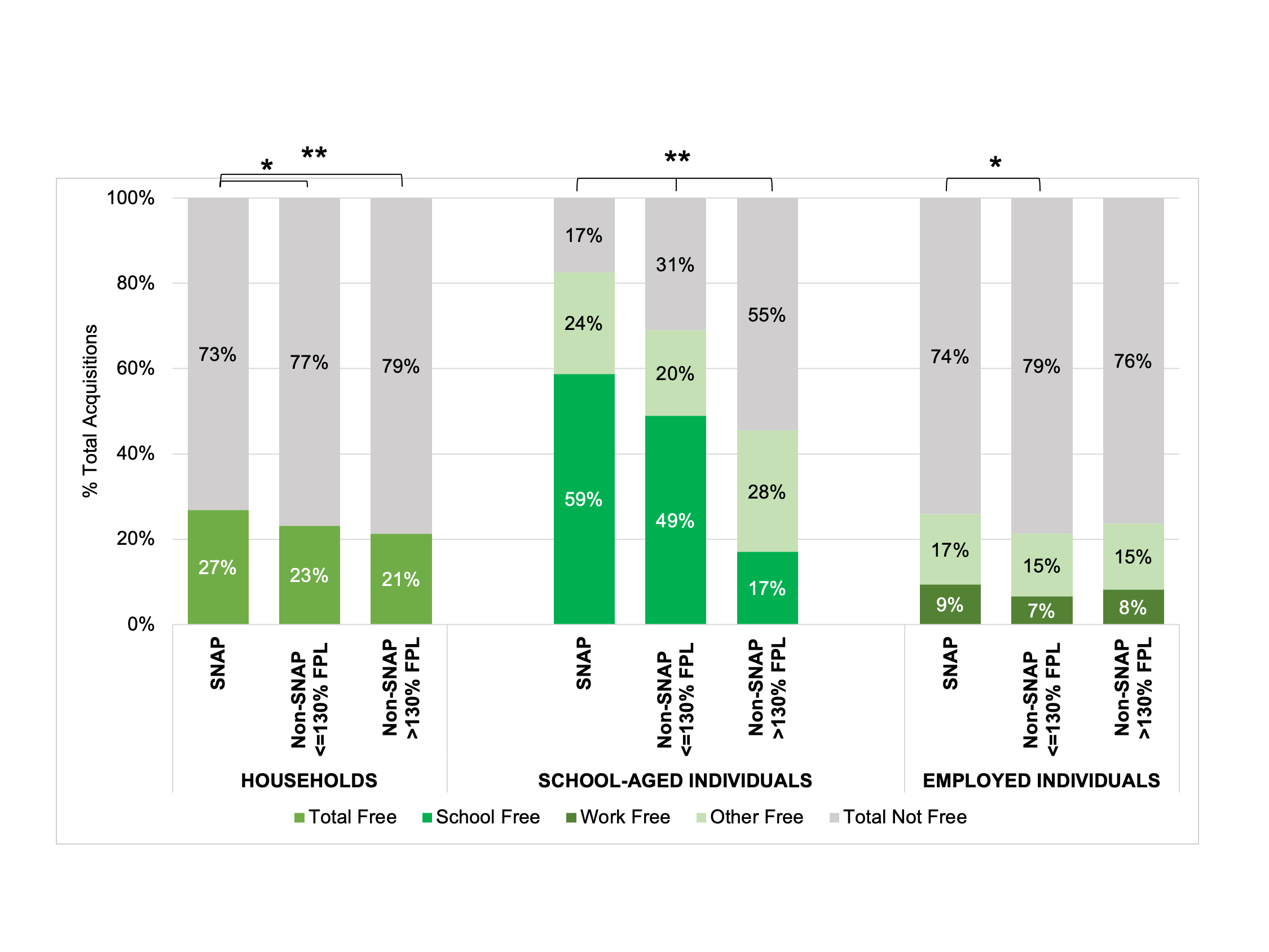

Supplement: S2 Fig — * % free significantly different between groups, p<0.05; ** p≤0.001. (TIF) [file pone.0257879.s002.tif]

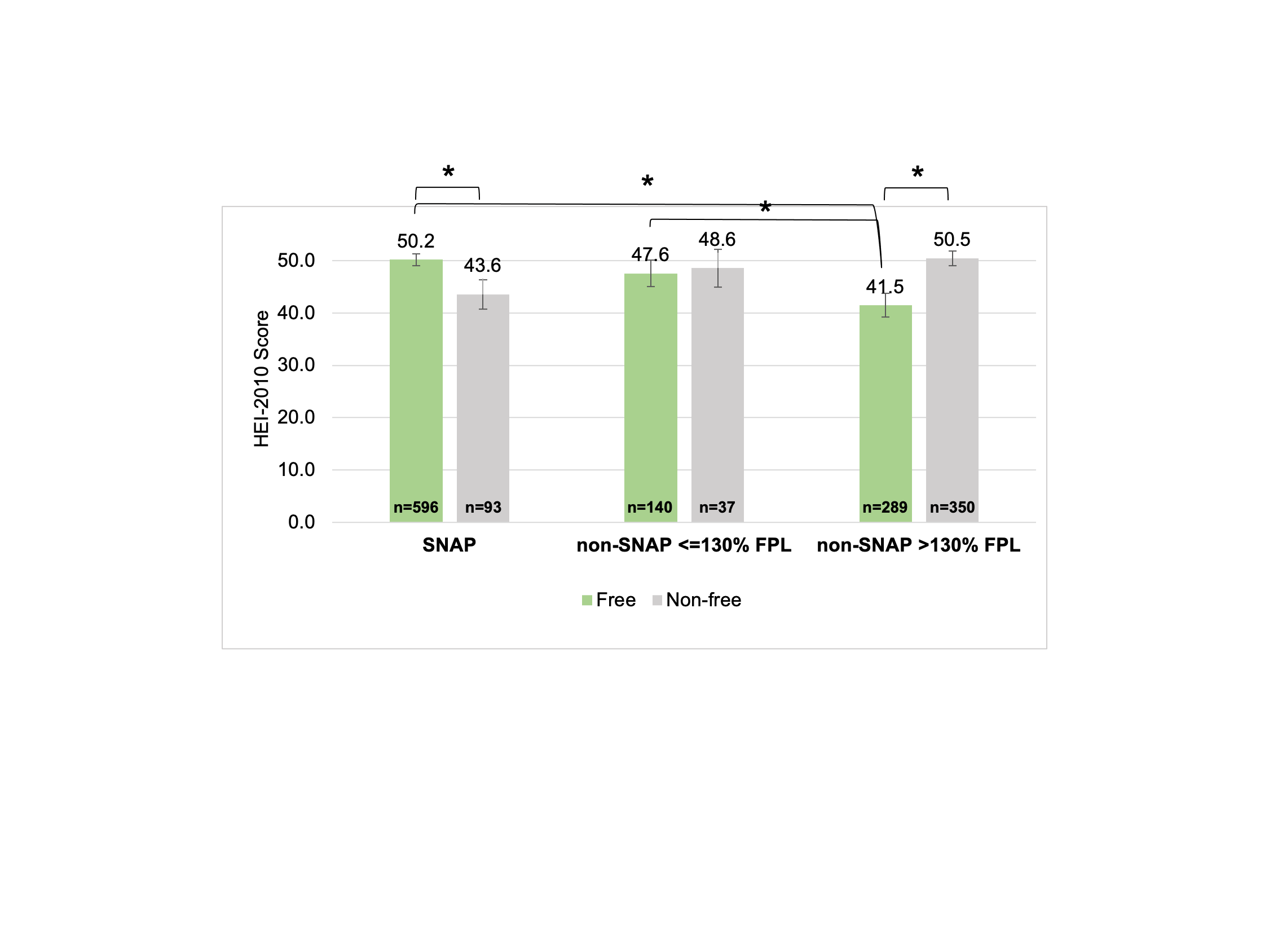

Supplement: S3 Fig — Survey-weighted, adjusted for individuals’ age, sex, race, Hispanic ethnicity, household number of children 5–18, household food insecurity, and household WIC status. *Sig. dif. p<0.05. (TIF) [file pone.0257879.s003.tif]

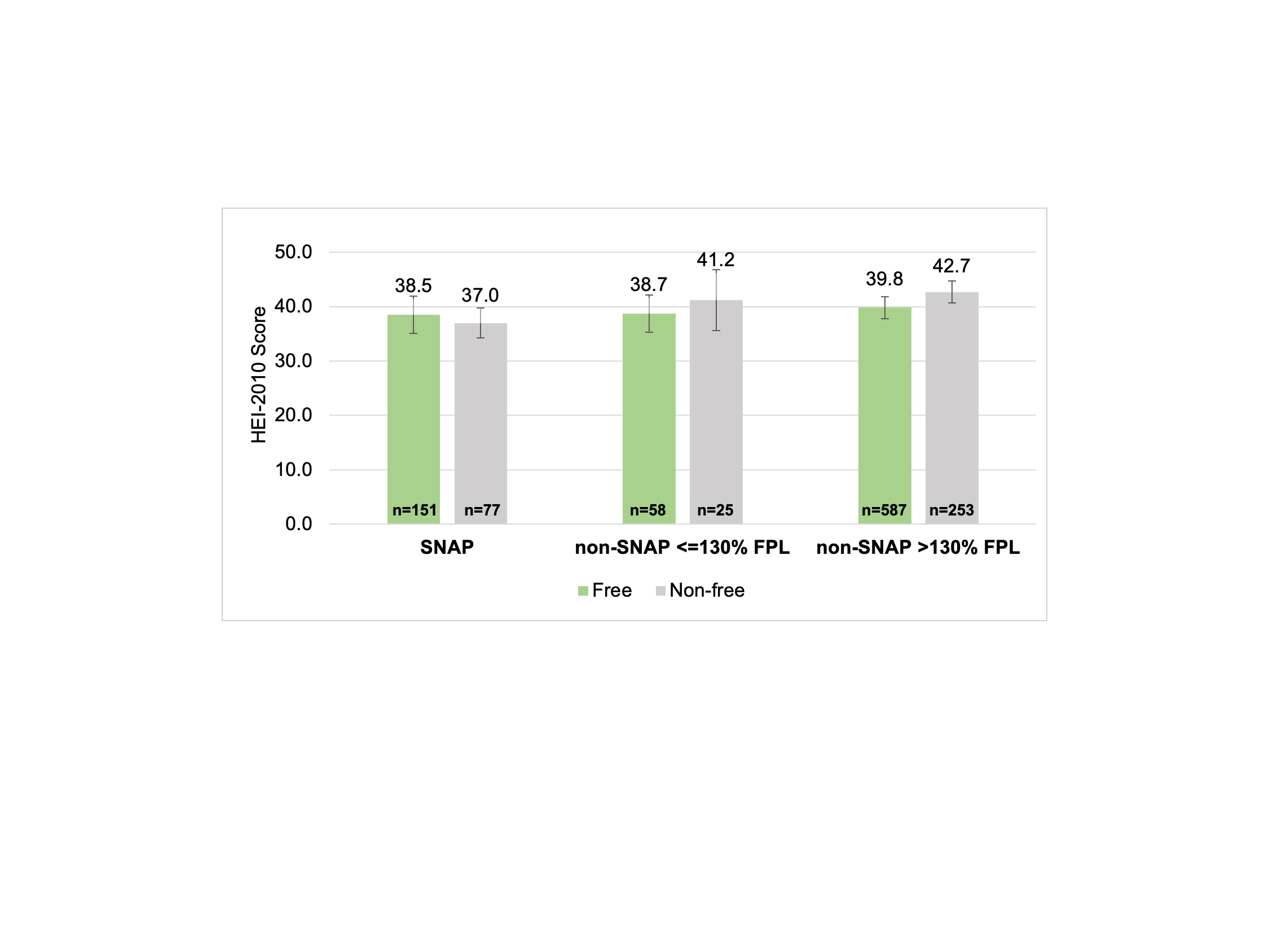

Supplement: S4 Fig — Survey-weighted, adjusted for individuals’ age, sex, race, Hispanic ethnicity, education, marriage status, household number of children 5–18, household food insecurity, and household WIC status. No significant differences. (TIF) [file pone.0257879.s004.tif]
