# Supplementary material for: Prevalence and nutritional quality of free food and beverage acquisitions at school and work by SNAP status
Source: PLoS One. 2021 Oct 13;16(10):e0257879. doi: 10.1371/journal.pone.0257879 (PMC8514130; doi:10.1371/journal.pone.0257879)
Supplement: S1 Table — Survey-weighted, adjusted for individuals’ age, sex, race, Hispanic ethnicity, household number of children 5–18, household food insecurity, and household WIC status. a Significantly different from SNAP individuals, p<0.05. b Calories from solid fats, alcohol, and added sugars; threshold for counting alcohol is >13 grams/1000 kcal. (DOCX) [file pone.0257879.s005.docx]

**S1 Table. HEI-2010 component density scores of free food acquisitions by children at school.**

|  | **Free School Acquisitions** | | |
| --- | --- | --- | --- |
|  | **SNAP individuals  (n=596)** | **non-SNAP <185% FPL individuals (n=247)** | **non-SNAP**  **>185% FPL individuals (n=182)** |
| **Component density scores (max score, standard for max score)** | **Mean (SE)** | **Mean (SE)** | **Mean (SE)** |
| Total vegetables (5, > 1.1 cups/1000kcal) | 2.38 (0.16) | 1.75 (0.22)^a^ | 0.96 (0.28)^a^ |
| Greens and beans (5, > 0.2 cups/1000kcal) | 0.92 (0.15) | 0.40 (0.12)^a^ | 0.05 (0.12)^a^ |
| Total fruit (5, > 0.8 cups/1000kcal) | 3.42 (0.17) | 2.56 (0.29)^a^ | 2.25 (0.39)^a^ |
| Whole fruit (5, > 0.4 cups/1000kcal) | 3.29 (0.18) | 2.38 (0.28)^a^ | 2.26 (0.44)^a^ |
| Whole grains (10, > 1.5 oz/1000kcal) | 2.56 (0.11) | 2.22 (0.18) | 2.51 (0.23) |
| Dairy (10, > 1.3 cups/1000kcal) | 8.75 (0.29) | 6.87 (0.65)^a^ | 7.45 (0.67) |
| Total protein foods (5, > 2.5 oz/1000kcal) | 3.74 (0.15) | 2.88 (0.32)^a^ | 2.05 (0.40)^a^ |
| Seafood and plant protein (5, > 0.8 oz/1000kcal) | 1.81 (0.17) | 1.30 (0.19) | 1.06 (0.25)^a^ |
| Fatty acids ratio (10, [PUFAS+MUFAS]/SFAs > 2.5) | 3.56 (0.29) | 2.80 (0.36) | 2.61 (0.51) |
| Sodium (10, < 1.1 grams/1000kcal) | 2.59 (0.25) | 2.44 (0.63) | 4.29 (0.76)^a^ |
| Refined grains (10, < 1.8 oz/1000kcal) | 3.19 (0.20) | 2.65 (0.52) | 2.90 (0.41) |
| Empty calories^b^ (20, < 19% of energy) | 12.94 (0.43) | 10.10 (0.95)^a^ | 9.44 (1.17)^a^ |

Survey-weighted, adjusted for individuals’ age, sex, race, Hispanic ethnicity, household number of children 5–18, household food insecurity, and household WIC status.

^a^ Significantly different from SNAP individuals, p<0.05.

^b^ Calories from solid fats, alcohol, and added sugars; threshold for counting alcohol is >13 grams/1000 kcal.
