# Supplementary material for: Prevalence and nutritional quality of free food and beverage acquisitions at school and work by SNAP status
Source: PLoS One. 2021 Oct 13;16(10):e0257879. doi: 10.1371/journal.pone.0257879 (PMC8514130; doi:10.1371/journal.pone.0257879)
Supplement: S2 Table — Survey-weighted, % out of total foods and beverages acquired for free at school by school-aged individuals. (DOCX) [file pone.0257879.s006.docx]

**S2 Table. Most commonly acquired foods and beverages for free by children at school.**

| **SNAP school-age individuals** | **%** | **non-SNAP <185% FPL** | **%** | **non-SNAP >185% FPL** | **%** |
| --- | --- | --- | --- | --- | --- |
| Fruits | 10.83 | Fruits | 10.91 | Milk | 9.81 |
| Milk | 10.75 | Milk | 10.79 | Fruits | 9.17 |
| Sandwiches | 10.16 | Sandwiches | 9.12 | Sandwiches | 8.56 |
| Flavored Milk | 10.09 | Flavored Milk | 8.41 | Flavored Milk | 8.48 |
| Vegetables (excluding potatoes) | 7.93 | Vegetables (excluding potatoes) | 8.33 | 100% Juice | 6.60 |
| 100% Juice | 7.65 | 100% Juice | 8.16 | Vegetables (excluding potatoes) | 6.21 |
| Pizza | 4.66 | White Potatoes | 4.46 | Sweet Bakery Products | 5.21 |
| White Potatoes | 3.42 | Pizza | 4.08 | White Potatoes | 5.00 |
| Quick Breads/Bread Products | 3.21 | Sweet Bakery Products | 3.11 | Pizza | 4.24 |
| Ready-to-Eat Cereals | 3.01 | Quick Breads/Bread Products | 2.86 | Poultry | 3.88 |

Survey-weighted, % out of total foods and beverages acquired for free at school by school-aged individuals.
