# Supplementary material for: Prevalence and nutritional quality of free food and beverage acquisitions at school and work by SNAP status
Source: PLoS One. 2021 Oct 13;16(10):e0257879. doi: 10.1371/journal.pone.0257879 (PMC8514130; doi:10.1371/journal.pone.0257879)
Supplement: S3 Table — Survey-weighted, % out of total foods and beverages acquired for free at school by school-aged individuals who do/don’t receive free school lunch. (DOCX) [file pone.0257879.s007.docx]

**S3 Table. Most commonly acquired foods and beverages for free by children at school, stratified by whether child receives free school lunch.**

| **All** | | | | **SNAP school-age individuals** | | | | **non-SNAP <185% FPL** | | | | **non-SNAP >185% FPL** | | | |
| --- | --- | --- | --- | --- | --- | --- | --- | --- | --- | --- | --- | --- | --- | --- | --- |
| **Free lunch** | **%** | **No free lunch** | **%** | **Free lunch** | **%** | **No free lunch** | **%** | **Free lunch** | **%** | **No free lunch** | **%** | **Free lunch** | **%** | **No free lunch** | **%** |
| Fruits | 10.92 | Sandwiches | 10.22 | Fruits | 11.12 | Milk | 14.13 | Fruits | 11.23 | 100% Juice | 9.82 | Milk | 11.81 | Flavored Milk | 10.18 |
| Milk | 10.84 | Milk | 9.07 | Flavored Milk | 10.55 | Sandwiches | 14.10 | Milk | 10.92 | Milk | 9.72 | Fruits | 9.93 | Sandwiches | 9.75 |
| Flavored Milk | 9.39 | Flavored Milk | 8.67 | Milk | 10.46 | Fruits | 7.48 | Sandwiches | 9.40 | Fruits | 8.40 | 100% Juice | 9.85 | Fruit | 8.32 |
| Sandwiches | 9.27 | Fruits | 8.17 | Sandwiches | 9.82 | 100% Juice | 7.41 | Vegetables (excluding potatoes) | 8.81 | Sandwiches | 6.94 | Sandwiches | 7.49 | Milk | 7.57 |
| Vegetables (excluding potatoes) | 8.22 | Sweet Bakery Products | 6.04 | Vegetables (excluding potatoes) | 8.26 | Sweet Bakery Products | 5.22 | Flavored Milk | 8.67 | Flavored Milk | 6.33 | Vegetables (excluding potatoes) | 7.35 | Sweet Bakery Products | 6.47 |
| 100% Juice | 8.16 | White Potatoes | 5.00 | 100% Juice | 7.67 | Flavored Milk | 4.70 | 100% Juice | 7.95 | Savory Snacks | 5.95 | Flavored Milk | 6.97 | White Potatoes | 5.81 |
| Pizza | 4.47 | Vegetables (excluding potatoes) | 4.70 | Pizza | 4.68 | Pizza | 4.52 | White Potatoes | 4.54 | Pizza | 5.70 | Pizza | 4.61 | Savory Snacks | 5.05 |
| White Potatoes | 3.89 | 100% Juice | 4.65 | White Potatoes | 3.47 | Mixed dishes (meat, poultry, seafood) | 4.41 | Pizza | 3.88 | Sweet Bakery Products | 4.95 | White Potatoes | 4.27 | Vegetables (excluding potatoes) | 4.93 |
| Ready-to-Eat Cereals | 3.05 | Savory Snacks | 4.64 | Quick Breads/Bread Products | 3.12 | Mixed dishes (Mexican) | 4.38 | Sweet Bakery Products | 2.88 | Vegetables (excluding potatoes) | 4.50 | Sweet Bakery Products | 4.09 | Poultry | 4.15 |
| Quick Breads/Bread Products | 3.04 | Pizza | 4.19 | Ready-to-Eat Cereals | 3.11 | Quick Breads/Bread Products | 4.22 | Quick Breads/Bread Products | 2.88 | White Potatoes | 3.79 | Ready-to-Eat Cereals | 4.07 | Candy | 3.97 |

Survey-weighted, % out of total foods and beverages acquired for free at school by school-aged individuals who do/don’t receive free school lunch.
