# Supplementary material for: Prevalence and nutritional quality of free food and beverage acquisitions at school and work by SNAP status
Source: PLoS One. 2021 Oct 13;16(10):e0257879. doi: 10.1371/journal.pone.0257879 (PMC8514130; doi:10.1371/journal.pone.0257879)
Supplement: S4 Table — Survey-weighted, adjusted for individuals’ age, sex, race, Hispanic ethnicity, education, marriage status, household number of children 5–18, household food insecurity, and household WIC status. a Significantly different from SNAP individuals, p<0.05. b Calories from solid fats, alcohol, and added sugars; threshold for counting alcohol is >13 grams/1000 kcal. (DOCX) [file pone.0257879.s008.docx]

**S4 Table. HEI-2010 component densities of free food acquisitions by employees at work.**

|  | **Free Work Acquisitions** | | |
| --- | --- | --- | --- |
|  | **SNAP employed individuals  (n=151)** | **non-SNAP <185% FPL (n=146)** | **non-SNAP >185% FPL (n=499)** |
| **Component density scores (max score, standard for max score)** | Mean (SE) | Mean (SE) | Mean (SE) |
| Total vegetables (5, > 1.1 cups/1000kcal) | 1.35 (0.34) | 1.56 (0.43) | 2.24 (0.23) |
| Greens and beans (5, > 0.2 cups/1000kcal) | 0.92 (0.46) | 1.05 (0.28) | 1.12 (0.27) |
| Total fruit (5, > 0.8 cups/1000kcal) | 0.84 (0.36) | 1.90 (0.56) | 0.69 (0.22) |
| Whole fruit (5, > 0.4 cups/1000kcal) | 0.63 (0.38) | 1.93 (0.60) | 0.71 (0.23) |
| Whole grains (10, > 1.5 oz/1000kcal) | 1.21 (0.40) | 1.35 (0.49) | 1.33 (0.38) |
| Dairy (10, > 1.3 cups/1000kcal) | 3.76 (0.71) | 3.32 (0.59) | 3.40 (0.46) |
| Total protein foods (5, > 2.5 oz/1000kcal) | 2.24 (0.50) | 2.32 (0.33) | 2.84 (0.29) |
| Seafood and plant protein (5, > 0.8 oz/1000kcal) | 1.08 (0.28) | 0.35 (0.31)^a^ | 1.24 (0.34) |
| Fatty acids ratio (10, [PUFAS+MUFAS]/SFAs > 2.5) | 5.27 (0.59) | 6.69 (0.57) | 4.99 (0.54) |
| Sodium (10, < 1.1 grams/1000kcal) | 3.92 (0.94) | 4.47 (0.83) | 3.78 (0.46) |
| Refined grains (10, < 1.8 oz/1000kcal) | 6.03 (0.96) | 6.00 (0.79) | 5.28 (0.56) |
| Empty calories^b^ (20, < 19% of energy) | 12.46 (1.88) | 10.40 (2.83) | 11.45 (1.28) |

Survey-weighted, adjusted for individuals’ age, sex, race, Hispanic ethnicity, education, marriage status, household number of children 5–18, household food insecurity, and household WIC status.

^a^ Significantly different from SNAP individuals, p<0.05.

^b^ Calories from solid fats, alcohol, and added sugars; threshold for counting alcohol is >13 grams/1000 kcal.
