# Supplementary material for: Prevalence and nutritional quality of free food and beverage acquisitions at school and work by SNAP status
Source: PLoS One. 2021 Oct 13;16(10):e0257879. doi: 10.1371/journal.pone.0257879 (PMC8514130; doi:10.1371/journal.pone.0257879)
Supplement: S5 Table — Survey-weighted, % out of total foods and beverages acquired for free at work by employed individuals. (DOCX) [file pone.0257879.s009.docx]

**S5 Table. Most commonly acquired foods and beverages for free by employees at work.**

| **SNAP employed individuals** | **%** | **non-SNAP <185% FPL** | **%** | **non-SNAP >185% FPL** | **%** |
| --- | --- | --- | --- | --- | --- |
| Coffee and tea | 14.16 | Coffee and tea | 9.85 | Coffee and tea | 23.30 |
| Sandwiches | 8.42 | Sandwiches | 8.76 | Sweetened beverages | 7.68 |
| Vegetables (excluding potatoes) | 7.78 | Vegetables (excluding potatoes) | 7.94 | Vegetables (excluding potatoes) | 5.98 |
| Sweetened beverages | 7.26 | Fruits | 7.15 | Sandwiches | 5.92 |
| Plain water | 5.74 | Plain water | 7.03 | Plain water | 5.66 |
| Quick breads/bread products | 4.42 | Sweetened beverages | 5.37 | Sweet bakery products | 5.40 |
| Breads, rolls, and tortillas | 3.82 | 100% juice | 5.21 | Breads, rolls, and tortillas | 4.07 |
| White potatoes | 3.50 | Sweet bakery products | 5.18 | Fruit | 3.14 |
| Poultry | 3.30 | Breads, rolls, and tortillas | 4.52 | Fats and oils | 3.12 |
| Fruits | 3.07 | Grain-based mixed dish | 3.59 | Diet beverages | 2.41 |

Survey-weighted, % out of total foods and beverages acquired for free at work by employed individuals.
